# Personality Traits and the Sense of Self-Efficacy among Nurse Anaesthetists. Multi-Centre Questionnaire Survey

**DOI:** 10.3390/ijerph18179381

**Published:** 2021-09-06

**Authors:** Magdalena Kwiatosz-Muc, Marzena Kotus, Anna Aftyka

**Affiliations:** Department of Anaesthesiological and Intensive Care Nursing, Medical University of Lublin, Chodzki Street 7, 20-093 Lublin, Poland; marzenakotus@umlub.pl (M.K.); anna.aftyka@umlub.pl (A.A.)

**Keywords:** general self-efficacy, personality traits, nurse anaesthetist

## Abstract

Introduction: Anaesthesia and intensive care units are specific workplaces. The purpose of this study was to evaluate the level of the sense of self-efficacy and the intensification of personality traits in a group of nurse anaesthetists and to develop a regression model explaining the sense of self-efficacy. Method: The population of the questionnaire survey included nurse anaesthetists from five hospitals in south-eastern Poland. The NEO-FFI was used in assessing their personality traits. The general self-efficacy scale was employed for the self-efficacy assessment. A total of 143 correctly filled surveys were analyzed. Results: The respondents typically perceived their own self-efficacy level as upper moderate. The nurse anaesthetists participating in the study revealed a tendency to high scores in conscientiousness and extraversion, and low scores related to neuroticism. The persons characterized by high conscientiousness, extraversion and openness to experience revealed a tendency to high scores related to the sense of self-efficacy. The relationship between personality traits and experiencing the nuisance of selected stressful job factors was demonstrated. Regression analysis showed that conscientiousness and extraversion are most closely related to the sense of self-efficacy. Conclusions: It seems to be beneficial to implement occupational consulting for nurses, who are starting their work or/and taking into consideration working in anesthesiology and intensive care units. The importance of personality traits and self- efficacy in relation with well-being of medical personnel needs deeper investigations.

## 1. Introduction

Post-graduate studies in anaesthesiology and intensive care in Poland are combined and nurses can specialise in anaesthesia and intensive care [1,2,3]. Anaesthesia and intensive care units are very specific workplace, where anaesthesiological teams supervise patients intensively for a long time and make interventions in critical situations [4]. The work is unpredictable and difficult to plan, often lasts many hours and takes place in a state of long-lasting physiological agitation. The duration of the work is also unpredictable and the breaks for hydration, eating or satisfying other physiological needs are rather irregular [5]. Nurse anaesthetists and intensive care nurses work in situations when urgent decisions are taken concerning critically ill patients’ treatment, which means the need for immediate performance of professional activities under severe stress. Complicated therapeutic procedures executed in patients in a life-threatening condition dominate in the work of the anaesthesia and intensive care unit staff. The staff operate complex equipment monitoring the patient’s condition and the devices which substitute the functions of failing organs, e.g., anaesthetic machines, ventilators, nephro-substitute treatment equipment, extracorporeal membrane oxygenation (ECMO) systems, etc. Moreover anaesthesia and intensive care unit doctors and nurses often contact patients whose lives are nearing the end [6].

Such stressful and heavy working conditions are a demanding environment for the staff and personality is among the crucial variables which affect the person’s adaptation and functioning in such a specific workplace. Personality is defined as a characteristic or regular way of responding to the social and natural environment and interacting with it. Costa and McCrae suggest that personality can be characterized as a set of basic dimensions or features meant as behavior properties, which reveal interindividual variability and intraindividual time and situation constancy [7]. Personality affects nearly every domain of human life—it is related to the sense of happiness, mental and physical health, spirituality and the quality of interpersonal relations, as well as the individual’s professional choices and political stance [8]. The personality model, according to Costa and McCrae, includes five main factors: neuroticism, extraversion, openness to experience, agreeableness, and conscientiousness. Numerous studies reveal that the intensification of the aforementioned personality dimensions in medical personnel is related to more severe stress, traumatic stress and burnout in this professional group [9,10]. Scientific research shows that neuroticism in health care staff coexisted with burnout [11,12,13], the level of traumatic stress [12] and fatigue [14]. Another personality dimension—agreeability—was related to burnout in the group of nurses working at the intensive neonatal care units and the level of traumatic stress in the staff. The research revealed that the strategies that reduce job-related stress might not be sufficient to reduce burnout and traumatic stress in nurses with a high level of neuroticism and a low level of agreeableness and extraversion [12].

Openness, extraversion, and conscientiousness seem to be correlated with a number of positive phenomena in the context of health care system functioning and the medical staff’s well-being. Openness and conscientiousness were related positively with job satisfaction [14]. Burgess et al. revealed the relationship between such personality dimensions as openness, agreeability and conscientiousness and coping with stress using problem-solving coping strategies. Openness and extraversion were related to a lower stress level caused by contacts with patients and their families. At the same time, conscientiousness was negatively correlated with the level of stress-related to overworking and stress resulting from a lack of self-confidence and competence [15]. Another study suggests a strong relationship between conscientiousness, agreeableness, openness, and neuroticism with the personal responsibility of nurses, who directly look after the patient. This observation is important because according to the results of the aforementioned study, high personal responsibility of nurses was related to a lower frequency of neglecting or postponing nursing care elements. The authors of the study point out that the nurse’s personality seems to be an essential condition of personal responsibility and, considering this during the nursing staff selection, can be useful in practice [16].

The sense of self-efficacy refers to an individual’s belief that they can take actions and reach the goal [17]. According to Albert Bandura’s concept, the perception of self-efficacy is related to the image of the individual’s competences and their providing of the measures that enable the performance of the intended actions [17]. A higher sense of self-efficacy improves motivation to act and is related to the individual’s greater accomplishments. The sense of self-efficacy may apply to one’s role in problematic and new situations [18]. Some studies reveal that the sense of self-efficacy is a vital factor in the analyses of the relationship between personality traits and experiencing stress [19]. The significance of the sense of self-efficacy (next to emotional intelligence and other psychological variables) in the prediction of some stress components intensification was demonstrated in a group of 1777 nurses [20].

The purpose of this study was to evaluate the intensification of personality traits and the level of the sense of self-efficacy in a group of nurse anaesthetists. The additional aim was to develop a regression model explaining the sense of self-efficacy.

## 2. Study and Methods

### 2.1. Sample Selection

This study was designed as a multicenter questionnaire survey. A total of 275 nurses working in anesthesiology and intensive care units (ICU) from five hospitals in south-eastern Poland were invited to the study. Investigations on personality traits and sense of self—efficacy in the group of medical personnel were conducted in different countries, however there are not known data concerning Poland. Hospitals were different sizes (academic centers and others). The research team personally, or via telephone, asked the heads of the chosen departments for their consent in participating in the research. The questionnaires were filled without the presence of the interviewer with the anonymity requirements fulfilled. The traditional form of a paper and pencil survey was used in the period of 1 May until 31 November 2019. Participants had no time limitation to fill the surveys. Consents were collected separately to preserve anonymity.

Inclusion criteria were as follows: employment in anesthesiology and intensive care unit for the last 6 months and giving written consent to participate in the study. In Poland, both doctors and nurses can specialize in anaesthesia and intensive care. The post-graduate studies in this area are combined. Nurse anaesthetists often take care for patients in operating theatres as well as in ICUs. In few hospitals nurse anaesthetists are employed only in ICU.

### 2.2. Methods

Paul Costa and Robert McCrae’s questionnaire called NEO-FFI was used in assessing the personality. The general self-efficacy scale (GSES) developed by Schwarzer and Jerusalem, adapted to the Polish conditions by Juczynski, was employed for the self-efficacy assessment. A proprietary survey on selected social and demographic characteristics (age, gender, marital status, place of living, having children, financial condition, type of hospital, unit, job seniority) as well as selected onerous (stressful) workplace factors were enclosed with the questionnaires. The proprietary survey included questions concerning the most onerous and stressful aspects of work in an anaesthesia and intensive care unit. It was constructed as a five-step Likert Scale (from 1 to 5). The study participant could indicate the middle of the scale (point 3 on the answers’ scale). The question was “How tiring are the following aspects of your job for you: working at night, the unpredictability of the work, being overloaded with duties, contact with death and complicated therapeutic procedures?”.

The general self-efficacy scale (GSES) is intended to measure the person’s general conviction about the efficacy of coping with difficult situations [18]. It is a short research tool consisting of 10 questions. The answers to the questions are arranged in a 4-point Likert scale. The scale’s overall score is the total number of points and gives the general indicator of the sense of self-efficacy, ranging from 10 to 40. The higher the GSES score, the higher the sense of self-efficacy is. After its conversion into standardised units, the obtained result is interpreted according to the properties characterising the sten score of ten. The scores between the first and the fourth sten are treated as low level, those between the seventh and the tenth sten as high ones. The scores between the fifth and the sixth sten score are regarded as medium. The reliability of the scale in the Polish language version is high (Cronbach’s alpha factor of 0.85). Theoretical accuracy was assessed comparing the results of the studies with few criteria related with personal competence idea [18].

The NEO-FFI is a personality questionnaire used by psychologists [21]. It measures five general personality dimensions according to the big five theory [21]. They include neuroticism (NEU), extraversion (EXT), openness to experience (OPN), agreeability (AGR) and conscientiousness (CON). The NEO-FFI consists of sixty statements which the respondent shall express their opinion about, according to the dedicated Likert scale. Every answer is pointed from 0 to 4 points, some questions in the scale are negatively constructed and some are positively constructed. For negative questions, it is necessary to change the points before summing them all up all points (0 = 4, 1 = 3, 2 remains unchanged, 3 = 1, 4 = 0). The overall result of the scale is the sum of all points (up to 48). Then, it is converted into a 10-degree standard ten score (called “sten score”). Conversion is based on tables with norms prepared separately for men and women divided into five age groups. Reliability of NEO-FFI scales was assessed with Cronbach’s alpha coefficient. The highest value of Cronbach’s alpha reached in CON Scale (0.82) and NEU Scale (0.80), lower in EXT Scale (0.77), and the lowest in OPN and ARG (0.68 both). Reliability coefficient for the Polish language version is lower than the original version, however it is acceptable for using the inventory for scientific research. Accuracy of NEO-FFI was assessed by verification of numerous hypotheses concerning relation between investigated traits with traits coherent with other conceptions of personality. Based on the test, some predictions can be formulated concerning the mind-set and approach to other people and the world and methods of coping with difficult situations [7,21]. Neuroticism means being susceptible to experiencing negative emotions such as fear, embarrassment, dissatisfaction, anger, sense of guilt and sensitivity to psychological stress. Regarding the fact that negative emotions affect an individual’s adaptation to the environment, neurotic persons are more prone to irrational ideas and have more difficulty coping with stress. Persons with low neuroticism level are emotionally stable, relaxed, and able to cope with the requirements of the environment, without experiencing any concerns, tension, or irritation. Extraversion is a dimension which characterises the quality and quantity of social interactions and the level of activity, energy, and ability to experience positive emotions. Extroverts are friendly and talkative but also eager to play and look for stimulation, and they demonstrate a positive mood. In turn, introverts are characterised by a lack of extravert behaviours rather than by their complete opposition. Openness to experience is a dimension which describes the individual’s proneness to look for and positively value life experiences, tolerance towards the new and cognitive curiosity. Agreeableness is a dimension which describes the attitude to other people and interpersonal orientation revealed in altruism. It stands for the trust of other people, being sensitive to other people’s issues and openness to cooperation. Conscientiousness is a dimension which characterises one’s level of being organised, persistent and motivated in goal-oriented actions. Persons with a high conscientiousness level reveal a strong will and are motivated to act and persist in pursuing their goals. They are also perceived as meticulous, punctual and reliable at work, and they have significant academic and professional accomplishments [7].

### 2.3. Approval of the Ethics Committee

The Ethics Committee of the Medical University of Lublin (KE-0254/267/2018) approved the study and all participants gave their written consent.

### 2.4. Statistical Methods

A statistical analysis of the study results was carried out using STATISTICA 13.3 PL statistical package.

Basic statistical measures, such as the mean (M), standard deviation (SD), median (Me) and 25–75% interquartile range, containing 50% of typical observations were used to describe the distribution of the GSES and NEO-FFI questionnaire values. The distribution of the size and percentage distribution of selected categories were given for the quality features.

The relationships’ analysis was carried out based on the Spearman’s rank correlation coefficient and t-test of the coefficient’s significance in the population.

Multiple regression models with demographic variables and intensification of personality traits, obtained in the NEO-FFI questionnaire, as the explanatory variables, were built to investigate the factors which significantly affect the sense of self-efficacy, expressed with the total score obtained in the GSES questionnaire.

The results significant on a typical significance level, i.e., when *p* ≤ 0.05, were considered statistically significant.

## 3. Results

The surveys were distributed among 275 persons. Only the correctly filled surveys (filled in accordance with the instructions and containing no missing items) were analysed (*n* = 143, 52%). The mean age of the respondents was nearly 45 years (44.99 ± 9.51). The great majority of the study population were women (*n* = 127, 88.8%), persons working in academic hospitals (*n* = 128, 89.5%) and persons with over ten years of job seniority (*n* = 116, 81.1%)—Table 1.

The study population members revealed upper medium scores in the GSES, which means that they evaluated their self-efficacy level as upper moderate. The mean raw score in the GSES amounted to 31.38 ± 3.98 points, whereas the mean sten score was 6.44 (SD 1.99). Nearly two-fifths of the study population reached the seventh sten score (*n* = 54, 37.8%).

The tendency to the low intensity of neuroticism was observed among the respondents; the mean sten score of the neuroticism intensity was 4.39 ± 2.0. The low intensity of the feature was observed in 78 respondents (54.4%). The study participants were prone to high extraversion (6.04 ± 1.76) and conscientiousness (6.43 ± 1.93)—high scores related to the intensity of the features were achieved by 59 (42.2%) and 71 (49.6%) respondents, respectively. The intensity of openness and agreeableness was moderate—Table 2 and Table 3.

The presence of statistically significant, negative correlation between the sense of self-efficacy and intensity of neuroticism (R = −0.30, *p* < 0.001) was demonstrated. Moreover, a significant positive correlation was discovered between the sense of self-efficacy and conscientiousness (R = 0.29, *p* < 0.001), extraversion (R = 0.29, *p* < 0.001) and openness to experience (R = 0.19, *p* < 0.05). No significant link between the sense of self-efficacy and agreeableness was observed—Table 4.

The analysis of the relationship between personality traits and experiencing the nuisance of selected stressful job factors revealed a positive correlation between the level of neuroticism and the level of the nuisance of the unpredictable nature of the job (R = 0.31, *p* < 0.001), complicated therapeutic procedures (R = 0.28, *p* < 0.001) and contact with death (R = 0.19, *p* < 0.05). A significant negative correlation was discovered between the level of extraversion with the nuisance of the unpredictable job nature (R = −0.20, *p* < 0.05) and being overloaded with duties (R = −0.22, *p* < 0.01). A significant positive correlation was also observed between the openness level and the nuisance of working at night (R = 0.26, *p* < 0.01) and being overloaded with duties (R = 0.17, *p* < 0.05). No significant correlation was discovered between the level of agreeableness and conscientiousness and experiencing the nuisance of the studied job factors.

The persons working only in the area of anaesthesiology and only in intensive care were separated from the whole study group. No differences were discovered between the intensity of the personality traits included in the NEO-FFI questionnaire and the declared place of work in the separated groups.

### Regression Analysis

A regression model was developed for the dependent variable of the self-efficacy level expressed as the GSES score and independent variables in the form of: gender, marital status and the intensity of personality traits. The regression model explains ca. 26% of the dependent variable value. The model is well adapted to the empirical data because the relative estimation error amounts to 11%. The independent variables in the model include extraversion, agreeableness, conscientiousness, gender, and marital status. Conscientiousness and extraversion are most closely related to the sense of self-efficacy and generally, the sense of self-efficacy increases as they go up. The sense of self-efficacy decreases with the increase in the level of agreeableness. The sense of self-efficacy is lower in persons involved in a relationship—Table 5.

An analogical regression model was carried out for women. It explains 22% of the dependent variable’s variability and renders a minor relative estimation error amounting to 11%. The dependent variable is significantly explained by extraversion, openness, and conscientiousness. No demographic variable was included in the model. Conscientiousness and extraversion are most closely related to the sense of self-efficacy. The increase in the variables usually entails a higher sense of self-efficacy—Table 6.

## 4. Discussion

The results achieved in this study seem to provide direct evidence which confirms the statement: in reference to such personality traits as conscientiousness, extraversion, and neuroticism the distribution of the respondents’ results significantly deviated from the normal distribution, characteristic of the general population. We do not have any results in this respect for the study population, but the reference literature indicates that the doctors, who specialize in emergency medicine differ significantly for their personality traits from doctors specializing in other fields of medicine [9]. The personality profile of emergency nurses differs from the population standard as well—in the studies carried out by Kennedy et al. their scores in the openness to experience, extraversion and agreeableness were higher than in the general population. The authors of the aforementioned research suggest that the assessment of personality and the knowledge of its influence on selecting the specialty can be helpful in improving the retention and recruitment of emergency nurses [22]. Burgess et al. suggest that some personality traits can turn into a buffer for stress at work. It seems that the screening tests before employing the staff, meant to identify the employees which reveal some personality traits, can be taken into consideration as part of recruitment strategy that could help to solve the problems related to the staff’s stress, diseases and retention [15].

The results obtained in this study, on the significance of personality traits in the perception of the level of such nuisance as working at night, unpredictable nature of the job, being overloaded with duties, contact with death and complicated therapeutic procedures require further studies. Burgess et al. demonstrated that openness and extraversion were related to a lower level of stress caused by the contacts with patients and their families. The same research’s results showed that conscientiousness was negatively correlated with the level of stress because of being overloaded with work, as well as stress resulting from a lack of self-confidence and competence [15]. The knowledge of psychological factors related to a lower sense of nuisance of job-related factors at a simultaneous high involvement and personal responsibility can be useful in the staff recruitment for nursing positions in the area of anaesthesiology and intensive care.

The hereby study population members revealed upper medium scores in the GSES, which means that they evaluated their self-efficacy level as upper moderate. The presence of statistically significant, negative correlation between the sense of self-efficacy and intensity of neuroticism was demonstrated. Moreover, a significant positive correlation was discovered between the sense of self-efficacy and conscientiousness, extraversion and openness to experience. The review of the reference literature provides only a few data related to the level, origin, and significance of the sense of self-efficacy in nurses, including nurse anaesthetists. It seems to be related to some personality dimensions, including neuroticism (negatively) and extraversion (positively) [23]. Better recognition of the factors which determine the sense of self-efficacy seems essential as it can be the factor which protects from stress and burnout [23]. The results obtained in this study, similarly to the results obtained by Yao et al. [23], emphasise the relationship between the sense of self-efficacy and extraversion and neuroticism, although the significance of the latter one disappears in the regression model. Culture factors can be playing an important role, but this hypothesis requires confirmation by scientific studies.

Poland has a unique place in the geographical and cultural map of Europe. Since 2003 Poland has been a member of the European Union (UE) and is closely related with Western Europe. However, by being the western border of UE is situated in the junction of western and eastern cultures. Historical and cultural functioning of the country could influence the functioning of its citizens including their occupational functioning. All of the studies concerning personality traits and general self-efficacy come from countries of UE or North America. Investigating this area in our country seems to be valuable.

### 4.1. Study Limitations

The majority of the study population are women, which is characteristic of nurses’ professional group in Poland. It would be productive to involve more males in the study. Unfortunately, it was not possible to include the adequately numerous group of males. For some time now, more males have been choosing the job of a nurse. In a few years, it will be possible to carry out such a study. The prevalence of females in nursing jobs has been described by many authors [23,24,25].

It shall be emphasized that the survey response rate in this study was low (52%), as compared to international standards. The low response rate of the survey questionnaires is common in Poland. Many Polish authors have shared the same observation.

We have to remember that in every survey study results reflect perceptions of participants. It should be taken into consideration in formulating any conclusions in survey studies. Self-efficacy and personality traits need further investigations with alternative methods to validate the findings of hereby study.

### 4.2. Theoretical Implications

It seems that in occupational context personality traits and general self-efficacy of examined nurses had similar importance in the studies carried out in Western civilization countries. The results of the studies need to be repeated. For the aim of future studies the hypothesis could be created that interventions functioning in western countries to strengthen psychological resources could be beneficial for polish nurses as well.

## 5. Conclusions

The respondents typically perceived their own self-efficacy level as upper moderate, revealed a tendency to high scores in conscientiousness and extraversion, and low scores related to neuroticism. The persons characterized by high conscientiousness, extraversion and openness to experience revealed a tendency to high scores related to the sense of self-efficacy. The relationship between personality traits and experiencing the nuisance of selected stressful job factors was demonstrated. For nurses with a high level of neuroticism working in anesthesiology and ICU could be more nuisance than for others. High levels of extraversion and openness seem to be related with lower experiencing of the nuisance of job occupation. It could be beneficial to implement occupational consulting for nurses, who are starting their work or/and taking into consideration working in anesthesiology and ICU. The importance of personality traits and self-efficacy in the relation with well-being of medical personnel needs deeper investigations. General self-efficacy as the potentially modified feature could be taken into consideration to be investigated in the future interventional studies reducing occupational stress level.

## Figures and Tables

**Table 1:** Group characteristics (*n* = 143).

Variables		N	%
Gender	Male	17	11.8
	Female	127	88.8
Type of hospital	Academic	128	89.5
	Other	16	11.2
Marital status	Married	99	69.2
	Unmarried	45	31.5
Place of living	Urban	114	79.7
	Rural	30	21.0
Job seniority	≤10 years	28	19.6
	>10 years	116	81.1
Unit	Anaesthesiology	68	47.6
	Anaesthesiology and intensive therapy	44	30.8
	Intensive therapy	31	21.7
Having children	Yes	103	72.0
	No	41	28.7
Financial condition	Satisfied	112	78.3
	Unsatisfied	32	22.4

**Table 2:** NEO-FFI results in sten scores.

Scales	NEO—FFI Sten Scores (*n* = 143)			
	M	SD	Me	25–75%
NEU	4.39	2.00	4.0	3.0–6.0
EXT	6.04	1.76	6.0	5.0–7.0
OPN	5.62	1.88	6.0	5.0–7.0
AGR	5.58	2.10	6.0	4.0–7.0
CON	6.43	1.93	6.0	5.0–8.0

NEU—neuroticism, EXT—extraversion, OPN—openness, AGR—agreeableness, CON—conscientiousness, M—mean, SD—standard deviation, Me—median.

**Table 3:** Percentage sten distribution in particular traits in the examined population.

Personality Trait		NEO—FFI: Sten Scores		
		Low (1–4)	Medium (5–6)	High (7–10)
NEU	*n* %	78 54.5	41 28.7	24 16.8
EXT	*n* %	30 21.0	54 37.8	59 41.2
OPN	*n* %	35 24.5	65 45.4	43 30.1
AGR	*n* %	41 28.7	62 43.3	40 28.0
CON	*n* %	24 16.8	48 33.6	71 49.6

NEU—neuroticism, EXT—extraversion, OPN—openness, AGR—agreeableness, CON—conscientiousness.

**Table 4:** Spearman’s rank correlation between general self-efficacy and personality traits intensity measured with NEO-FFI in sten score.

Pair of Variables	N	R	Test t
NEU and GSE	143	−0.30 ***	−3.743
EXT and GSE	143	0.29 ***	3.564
OPN and GSE	143	0.19 *	2.287
AGR and GSE	143	−0.00	−0.034
CON and GSE	143	0.29 ***	3.606

NEU—neuroticism, EXT—extraversion, OPN—openness, AGR—agreeableness, CON—conscientiousness, GSE—general self-efficacy, R—Spearman’s coefficient, * *p* < 0.05, *** *p* < 0.001.

**Table 5:** Regression analysis for dependent variable general self-efficacy.

Variable	B	SE b	Beta	SE Beta	t (137)	*p*
(Constant)		Variable	26.46	1.442	18.34	0
EXT	0.257	0.078	0.58	0.175	3.31	0.001
AGR	−0.182	0.082	−0.35	0.155	−2.23	0.028
CON	0.306	0.081	0.63	0.168	3.77	0
Gender (1 = male)	0.192	0.074	2.41	0.936	2.58	0.011
Marital status (1 = married)	−0.174	0.074	−1.49	0.633	−2.35	0.02

EXT—extraversion, AGR—agreeableness, CON—conscientiousness, b—unstandardized regression weights, beta—standardized regression weights, SE—statistical error.

**Table 6:** Regression analysis in the female group for dependent variable general self-efficacy.

Variable	b	SE b	Beta	SE Beta	t (123)	*p*
(Constant)			22.38	1.523	14.69	0
EXT	0.235	0.085	0.52	0.19	2.77	0.006
OPN	0.174	0.083	0.37	0.177	2.08	0.04
CON	0.253	0.083	0.53	0.173	3.05	0.003

EXT—extraversion, OPN—openness, CON—conscientiousness. b—unstandardized regression weights, beta—standardized regression weights, SE—statistical error.

## Data Availability

The data that support the findings of this study are available from the corresponding author, [MKM], upon reasonable request.
