# Correction: Kwiatosz-Muc et al. Personality Traits and the Sense of Self-Efficacy among Nurse Anaesthetists. Multi-Centre Questionnaire Survey. *Int. J. Environ. Res. Public Health* 2021, *18*, 9381

**DOI:** 10.3390/ijerph182111118

**Published:** 2021-10-22

**Authors:** Magdalena Kwiatosz-Muc, Marzena Kotus, Anna Aftyka

**Affiliations:** Department of Anaesthesiological and Intensive Care Nursing, Medical University of Lublin, Chodzki Street 7, 20-093 Lublin, Poland; marzenakotus@umlub.pl (M.K.); anna.aftyka@umlub.pl (A.A.)

## Text Correction

There was an error in the original article [1]. The great majority of the study population were women (*n* = 127, 88.8%), persons working in academic hospitals (*n* = 128, 89.5%) and persons with over ten years of job seniority (*n* = 116, 81.1%)—Table 1.

A correction has been made to Results, Paragraph 1:

The great majority of the study population were women (*n* = 127, 88.8%), persons working in academic hospitals (*n* = 127, 88.8%) and persons with over ten years of job seniority (*n* = 115, 80.4%)—Table 1.

The authors apologize for any inconvenience caused and state that the scientific conclusions are unaffected. The original article has been updated.

## Incorrect Funding Number

There is an error in the Funding statement. The correct number for Medical University of Lublin is Grant Number DS 548. The authors apologize for any inconvenience caused and state that the scientific conclusions are unaffected. The original article has been updated.

## Error in Table

In the original article, there was a mistake in Table 1 as published. Several numerical mistakes were made.

**Table 1:** Group characteristics (*n* = 143).

Variables		N	%
Gender	Male	17	11.8
	Female	127	88.8
Type of hospital	Academic	128	89.5
	Other	16	11.2
Marital status	Married	99	69.2
	Unmarried	45	31.5
Place of living	Urban	114	79.7
	Rural	30	21.0
Job seniority	≤10 years	28	19.6
	>10 years	116	81.1
Unit	Anaesthesiology	68	47.6
	Anaesthesiology and intensive therapy	44	30.8
	Intensive therapy	31	21.7
Having children	Yes	103	72.0
	No	41	28.7
Financial condition	Satisfied	112	78.3
	Unsatisfied	32	22.4

The corrected Table 1 appears below. The authors apologize for any inconvenience caused and state that the scientific conclusions are unaffected. The original article has been updated.

**Table 1:** Group characteristics (*n* = 143).

Variables		N	%
Gender	Male	16	11.2
	Female	127	88.8
Type of hospital	Academic	127	88.8
	Other	16	11.2
Marital status	Married	98	68.6
	Unmarried	45	31.4
Place of living	Urban	113	79.0
	Rural	30	21.0
Job seniority	≤10 years	28	19.6
	>10 years	115	80.4
Unit	Anesthesiology	68	47.6
	Anesthesiology and intensive therapy	44	30.7
	Intensive therapy	31	21.7
Having children	Yes	102	71.3
	No	41	28.7
Financial condition	Satisfied	111	77.7
	Unsatisfied	32	22.3
